# The correlation and role analysis of COL4A1 and COL4A2 in hepatocarcinogenesis

**DOI:** 10.18632/aging.102610

**Published:** 2020-01-05

**Authors:** Yanli Liu, Jiaye Zhang, Yan Chen, Hasan Sohel, Xinrong Ke, Jingqi Chen, Yin-Xiong Li

**Affiliations:** 1 Stem Cell Translational Medicine Center, State Key Laboratory of Respiratory Disease, The Second Affiliated Hospital, Guangzhou Medical University, Guangzhou, China; 2 Stem Cell Translational Medicine Center, The Second Affiliated Hospital, Guangzhou Medical University, Guangzhou, China; 3 Department of Medical Oncology, Guangzhou Medical University, Guangzhou, China; 4 Institute of Public Health, Guangzhou Institutes of Biomedicine and Health, Chinese Academy of Sciences, Guangzhou, China; 5 Key Laboratory of Regenerative Biology, South China Institute for Stem Cell Biology and Regenerative Medicine, Guangzhou Institutes of Biomedicine and Health, Chinese Academy of Sciences, Guangzhou, China; 6 Guangdong Provincial Key Laboratory of Biocomputing, Guangzhou Institutes of Biomedicine and Health, Chinese Academy of Sciences, Guangzhou, China; 7 Guangdong Provincial Key Laboratory of Stem Cell and Regenerative Medicine, Guangzhou, China; 8 University of Chinese Academy of Sciences, Beijing, China

**Keywords:** tumor microenvironment, COL4A1/A2, HCC, PI-3K pathway, PTK2

## Abstract

Liver fibrosis biomarker, Type IV collagen, may function as hepatocarcinogenesis niche. However, among the six isoforms, the isoforms providing tumor microenvironment and their regulatory network are still unclarified. Based on bioinformatics analysis of hundreds of HCC transcriptome datasets from public databases, we found that *COL4A1/2* expressions were significantly correlated with hepatocarcinogenesis, progression, and prognosis. The expressions of *COL4A1/2* were significantly upregulated in the preneoplastic and HCC tissues compared with normal tissues. Moreover, the overexpression of *COL4A2* was highly correlated with shorter progression-free survival in HCC patients. Bioinformatics analysis also generates an interactive regulatory network in which COL4A1/2 directly binding to integrin alpha-2/beta-1 initiates a sequentially and complicated signaling transduction, to accelerate cell cycle and promote tumorigenesis. Among those pathways, the PI3K-Akt pathway is significantly enriched in cooperative mutations and correlation analysis. This suggests that the key activated signaling is PI3K-Akt pathway which severing as the centerline linked with other pathways (Wnt and MAPK signaling) and cell behaviors signaling (cell cycle control and cytoskeleton change). Switching extracellular matrix collagen isoform may establish pro-tumorigenic and metastatic niches. The findings of *COL4A1/2* and related signaling networks are valuable to be further investigated that may provide druggable targets for HCC intervention.

## INTRODUCTION

Hepatocellular carcinoma (HCC) is the most prevalent malignancy in the liver with a high incidence and mortality rate globally [1]. It is the fifth most commonly diagnosed cancer in men, the ninth in women, and the second leading cause of cancer death worldwide in 2012 [2]. HCC had a poor prognosis as the ratio of mortality to incidence is 0.95 [2] and the 5-year survival rate of advanced HCC was less than 5% [3]. The advanced fibrosis, mainly cirrhosis and hepatitis, caused by the Hepatitis B virus (HBV) and hepatitis C virus (HCV) were the most common cause for HCC [4–6]. Moreover, other factors such as alcohol consumption, obesity, aflatoxin exposure, and nonalcoholic fatty liver disease could also contribute to the development of HCC [4]. The understanding of the etiology and the molecular mechanisms of HCC leads to the development of drugs including sorafenib, regorafenib, nivolumab, and lenvatinib, which have been approved by the FDA in unresectable HCC treatment [7–10]. However, these drugs are not satisfying, due to the rapid drug resistance development and toxicities [9, 11–13]. Therefore, there is an urgent need for further understanding of the pathological mechanism of HCC and developing combination therapies that target different signaling mechanisms to reduce the drug resistance of HCC treatment.

The tumor microenvironment is an important part of tumor structure and function, and it plays a key role in the initiation and progression of carcinogenesis [14, 15]. Therefore, a better understanding of the molecular mechanism of the tumor microenvironment may provide new and valuable targets for cancer prevention, management, and risk assessment. As the major structural component of the tumor microenvironment, type IV collagen (Col IV) forms a ‘chicken-wire’ meshwork together with laminins, proteoglycans and entactin/nidogen. Also, Col IV has been found as a useful marker for evaluating liver preneoplastic lesions (fibrosis and cirrhosis), for its swift increase with fibrotic progression [16–18]. Moreover, the Col IV serum level was found to be a marker for evaluating primary and metastatic liver cancer, and recurrence risk of HCC after liver resection [19]. Recently, Col IV has shown the ability to promote liver metastasis of lung cancer by regulating the chemokine CCL7 signals [20, 21]. Therefore, Col IV may play a key role in hepatocarcinogenesis. However, the underlying mechanism still needs to be elucidated. Moreover, there are six Col IV isoforms, α1 (IV)-α6 (IV) which are encoded by *COL4A1*-*COL4A6* genes, and the regulatory role of each isoform in HCC has yet to be discovered.

To address the above questions, the sequencing data of HCC were collected and analyzed from GEO and TCGA databases. The result showed that among the six Col IV isoforms, only *COL4A1* and *COL4A2* were significantly upregulated from liver preneoplastic lesions (cirrhosis and dysplasia) to HCC. Subsequently, the *COL4A1* and *COL4A2* network genomic alterations, biological function, and regulatory network in HCC were analyzed by using cBioPortal and LinkedOmics. Thus, this study revealed the expression and regulatory network of *COL4A1* and *COL4A2* in hepatocarcinogenesis, which might be novel targets for HCC diagnosis and treatment.

## RESULTS

### Transcriptional levels of COL4As in the carcinogenic process from preneoplastic lesions (cirrhosis and dysplasia) to HCC

**Figure 1:**
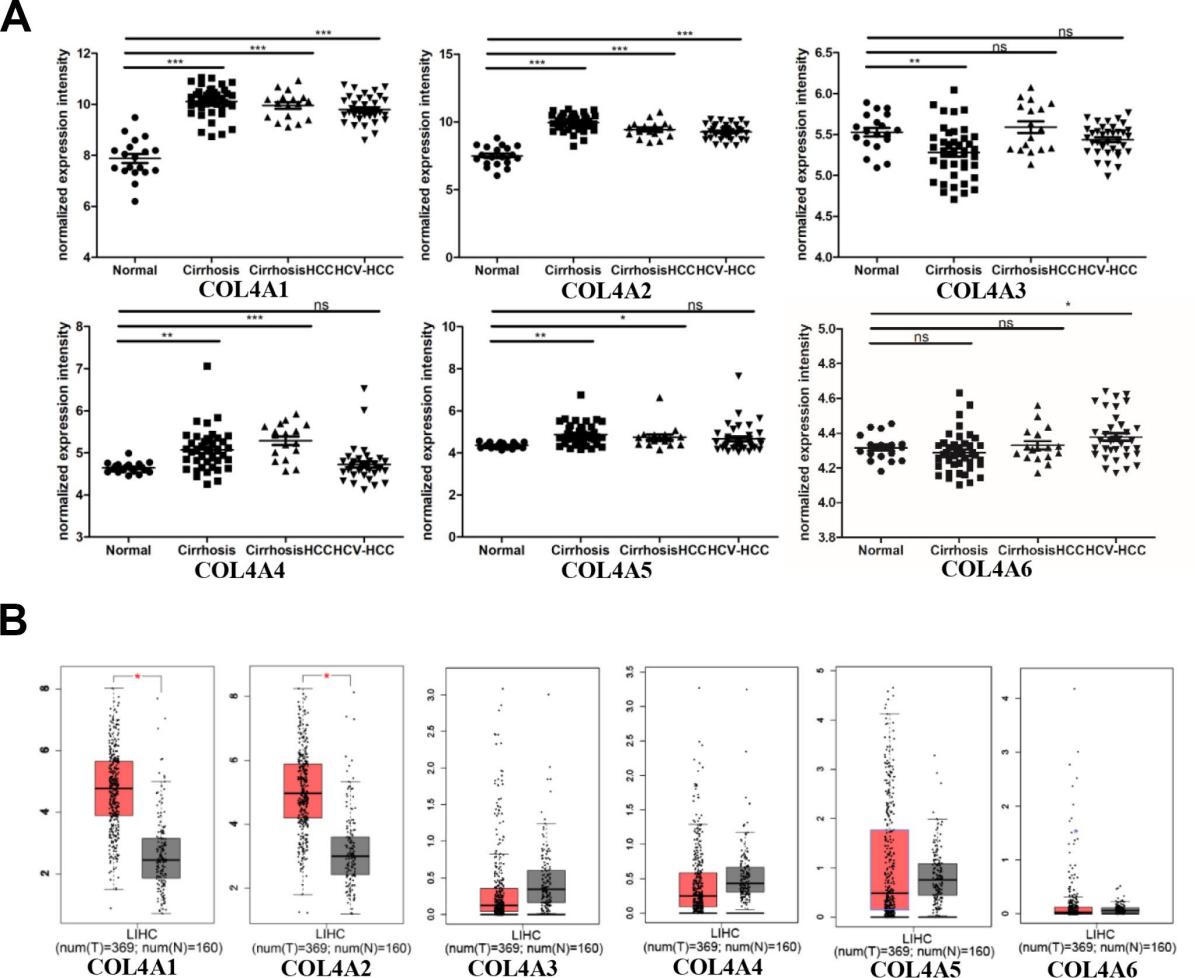
**COL4As expression in hepatocellular carcinoma (HCC).** The transcription levels of *COL4A1* and *COL4A2* were significantly upregulated in preneoplastic lesion (cirrhosis) and HCC tissues compared to normal tissues. (**A**) Dot plot showing the COL4As mRNA levels in GEO dataset (GSE14323). Normal (n=19): normal liver; Cirrhosis (n=41): HCV cirrhosis without HCC; Cirrhosis HCC (n=17): HCV cirrhosis with HCC; HCV-HCC (n=36): HCC by HCV infection. Data are mean ± SD. *, P < 0.05, **, P < 0.01, ***, P < 0.001 (Student’s t-test). (**B**) Box plot showing the COL4As mRNA levels in The Cancer Genome Atlas (TCGA) (GEPIA). Normal: n=160; Tumor: n=369. The significance test method was one-way ANOVA, using disease state (Tumor or Normal) as variable for calculating differential expression.

**Figure 2:**
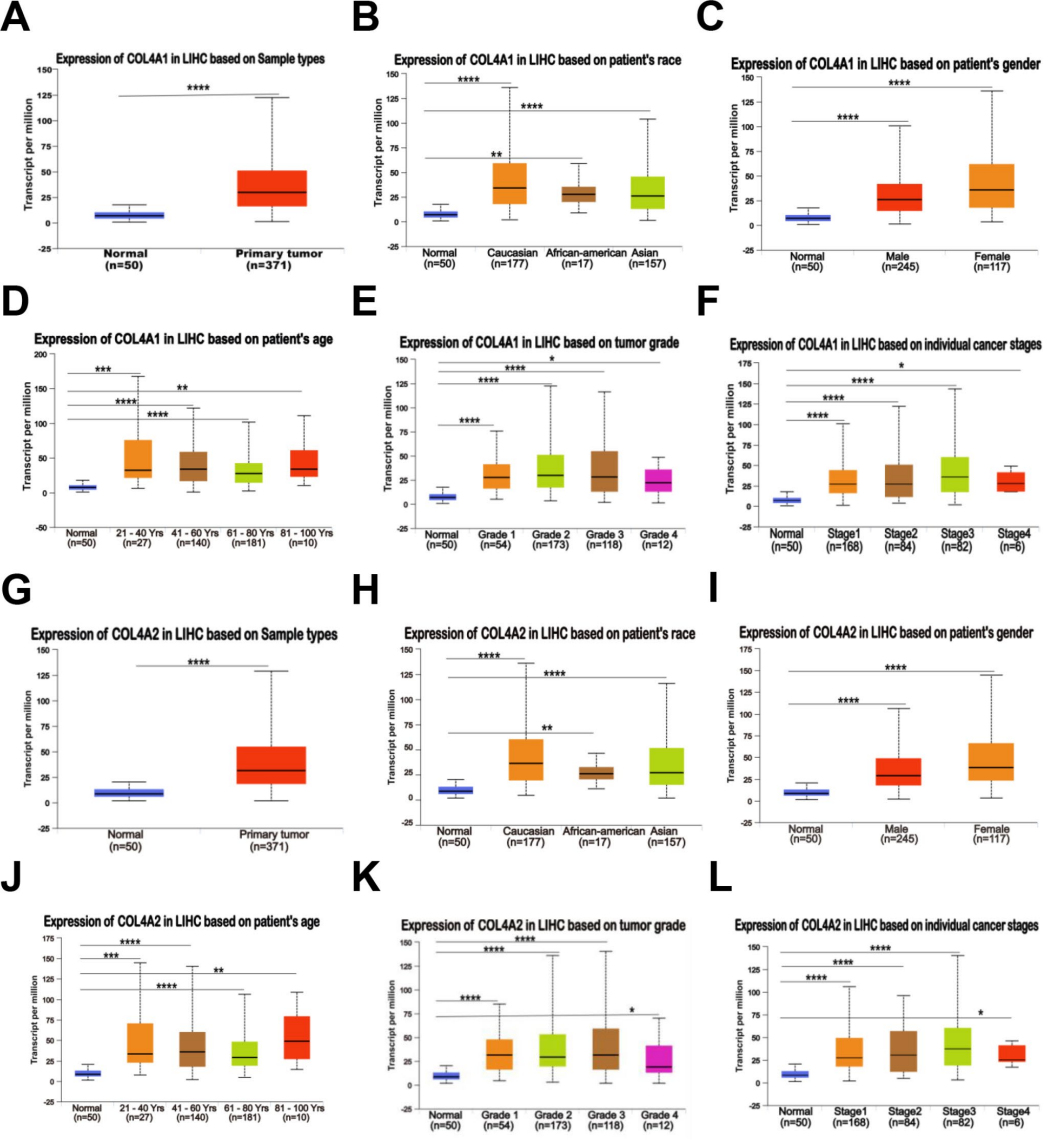
**Relationship between the mRNA levels of *COL4A1*/*2* and the clinic pathological features of patients with HCC, stratified based on ethnicity, gender, age, tumor grade, and disease stages (UALCAN).** (**A**, **G**) Box plot showing the relative transcript level of *COL4A1* and *COL4A2* in normal and primary tumor (HCC) tissues. (**B**, **H**) Box plot showing the relative transcript level of *COL4A1* and *COL4A2* in normal samples of any ethnicity, and HCC tissues of Caucasian, African-American or Asian. (**C**, **I**) Box plot showing the relative transcript level of *COL4A1* and *COL4A2* in normal tissues of any gender, and HCC tissues of male or female. (**D**, **J**) Box plot showing the relative transcript level of *COL4A1* and *COL4A2* in healthy individuals of any age, and HCC tissues of 21–40, 41–60, 61–80, or 81–100 yrs. (**E**, **K**) Box plot showing the relative transcript level of *COL4A1* and *COL4A2* in normal tissues, and HCC tissues with grade 1, 2, 3 or 4. (**F**, **L**) Box plot showing the relative transcript level of *COL4A1* and *COL4A2* in normal tissues, and HCC tissues in stage 1, 2, 3 or 4. Data are mean ± SE. *, P < 0.05; **, P < 0.01; ***, P < 0.001, ****, P < 0.0001 (t-test).

**Figure 3:**
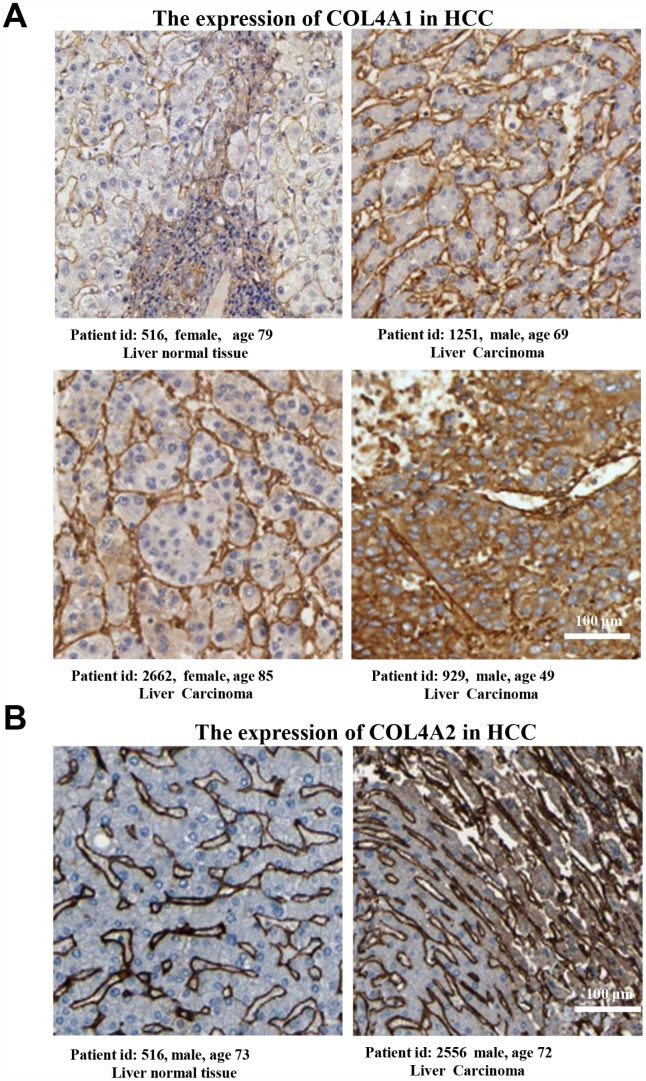
**The COL4A1 and COL4A2 proteins were expressed higher in HCC tissues than in the normal liver tissue.** (**A**–**B**) Immunohistochemistry staining showing the protein expression of COL4A1 (**A**) and COL4A2 (**B**) in liver normal tissues and liver carcinoma.

Six COL IV isoforms (COL4As) have been identified in mammalian cells. We initially assessed the transcriptional levels of COL4As in multiple HCC studies from GEO and TCGA databases. The mRNA expression levels of *COL4A1* and *COL4A2* were significantly upregulated in patients with liver cirrhosis and HCC tissues in two datasets. In the Mas Liver (GSE14323), *COL4A1* was overexpressed in liver cirrhosis (fold change = 4.233, p = 2.78E-13) and HCC (fold change =3.759, p = 1.40E-12), while *COL4A2* was higher expressed in liver cirrhosis (fold change = 2.487, p = 7.88E-14) and HCC (fold change =3.492, p = 1.01E-10) versus normal tissues (Figure 1A and Supplementary Figure 1B). In the Wurmbach liver (GSE6764), *COL4A1* was increased in cirrhosis (fold change =2.997, p = 7.24E-6), liver cell dysplasia (fold change =2.140, p = 7.46E-6), and HCC (fold change =3.711, p = 1.16E-10). *COL4A2* was also increased in cirrhosis (fold change =3.412, p = 2.02E-6), liver cell dysplasia (fold change =2.223, p = 1.35E-4), and HCC (fold change =3.154, p = 7.07E-7) compared to normal tissues (Supplementary Figure 1A and 1C). Apart from this, *COL4A1* and *COL4A2* were in the top 5% over-expression gene rank of liver cirrhosis and HCC in both datasets (Supplementary Figure 1B, 1C). In comparison, *COL4A3*-*COL4A6* were not significantly changed in HCC versus normal tissues (Figure 1A and Supplementary Figure 1A). Further analysis of 371 HCC patients in TCGA consistently showed different effects of *COL4A1*-*COL4A6* in hepatocarcinogenesis (Figure 1B). Moreover, the mRNA levels of both COL4A1 and COL4A2 were significantly increased in subgroups of HCC patients classified by ethnicity, gender, age, tumor grade, and disease stages compared to normal people (Figure 2A–2L). Additionally, the expressions of COL4A1 and COL4A2 in HCC and normal individuals were evaluated by immunohistochemistry staining (The Human Protein Atlas). The COL4A1 and COL4A2 proteins were more highly expressed in HCC tissues than in the normal liver tissues, and were located especially in the HCC tissue lacunar (Figure 3). Thus, *COL4A1* and *COL4A2* expressions may serve as potential diagnostic indicators in HCC.

### The cooperative genomic alterations of *COL4A1* and *COL4A2* networks in HCC

We then analyzed the cooperative genomic alterations of *COL4A1* and *COL4A2* networks by using cBioPortal for liver hepatocarcinoma (TCGA, provisional).

**Figure 4:**
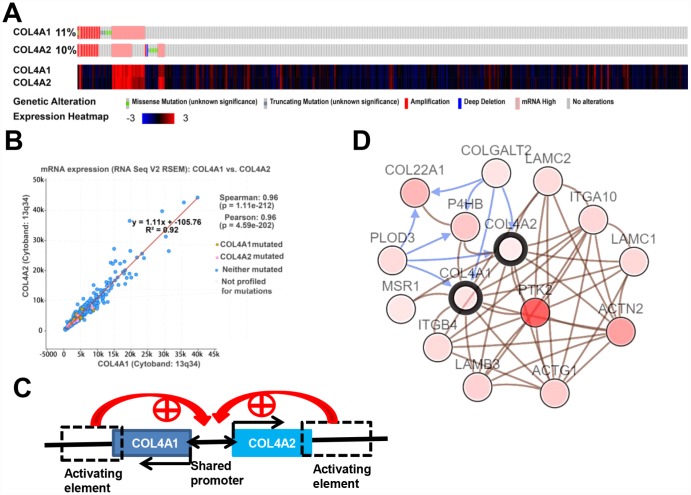
***COL4A1* and *COL4A2* genomic alterations, correlation, and signaling pathway in HCC (cBioPortal).** (**A**) Heat map showing the genomic alteration types and expression of *COL4A1* and *COL4A2* in HCC tissues (n=371). The genomic alterations are highlighted in different markers. One column represents one individual. (**B**) Correlation analysis showing the significant positive correlation between *COL4A1* and *COL4A2*. (**C**) *COL4A1* and *COL4A2* shared a bidirectional promoter, which was regulated by the downstream activating element. (**D**) Network showing the *COL4A1*/*COL4A2* (with a bold border) and their neighboring genes with alteration frequencies >10% in HCC. *PTK2* (in darker red) was the most frequently altered neighbor gene (46.4%). The brown connection shows that these genes are involved in the same biological component, such as a complex. The blue connection shows that the first gene causes a state change, such as a phosphorylation change, within the second gene.

*COL4A1* was altered in 41 of 371 (11%) HCC patients, and *COL4A2* was altered in 37 of 371 (10%) HCC patients (Figure 4A). The genomic alterations included amplification (3.8% for both *COL4A1* and *COL4A2*), mRNA up-regulation (5.6% for *COL4A1*, 4.6% for *COL4A2*), and mutation (2.1% for *COL4A1*, 1.6% for *COL4A2*) (Table 1). Thus, mRNA up-regulation and amplification are the most genomic alteration types for both *COL4A1* and *COL4A2* in HCC.

**Table 1:** The most frequently altered neighbor genes of *COL4A1* and *COL4A2* in HCC(cBioPortal).

**Gene symbol**	**Amplification**	**Homozygous deletion**	**Up-regulation**	**Down-regulation**	**Mutation**	**Total alteration**
COL4A1	3.80%	0.00%	5.60%	0.00%	2.10%	11.00%
COL4A2	3.80%	0.30%	4.60%	0.00%	1.60%	9.90%
PTK2	16.10%	0.30%	41.00%	0.50%	2.10%	46.40%
ACTN2	8.60%	0.00%	15.50%	0.00%	1.60%	24.10%
THBS3	13.70%	0.00%	13.70%	0.00%	1.60%	20.10%
COL22A1	16.40%	0.00%	1.10%	0.00%	3.20%	19.30%
P4HB	6.20%	0.30%	11.80%	0.30%	0.30%	15.80%
ACTG1	6.20%	0.00%	9.90%	0.00%	0.30%	15.30%
LAMB3	9.40%	0.00%	3.50%	0.00%	1.90%	13.90%
ITGA10	9.90%	0.00%	2.10%	0.00%	1.90%	13.10%
LAMC1	9.10%	0.00%	3.20%	0.00%	1.60%	12.90%
LAMC2	9.10%	0.00%	1.90%	0.00%	1.60%	12.60%
ITGB4	5.60%	0.00%	4.80%	0.00%	2.40%	12.60%
PLOD3	0.80%	0.00%	11.50%	0.50%	0.30%	12.60%
COLGALT2	9.40%	0.00%	0.50%	0.00%	1.30%	11.00%
MSR1	0.30%	7.00%	3.50%	0.00%	0.30%	10.20%

Moreover, the expression levels of *COL4A1* and *COL4A2* were highly correlated in 371 HCC patients (Figure 4A). Therefore, the correlation coefficient between *COL4A1* and *COL4A2* in HCC was calculated via cBioPortal. The results showed that *COL4A1* was strongly correlated with *COL4A2* (r = 0.96), possibly because they shared the same bidirectional promoter (Figure 4B, 4C).

**Figure 5:**
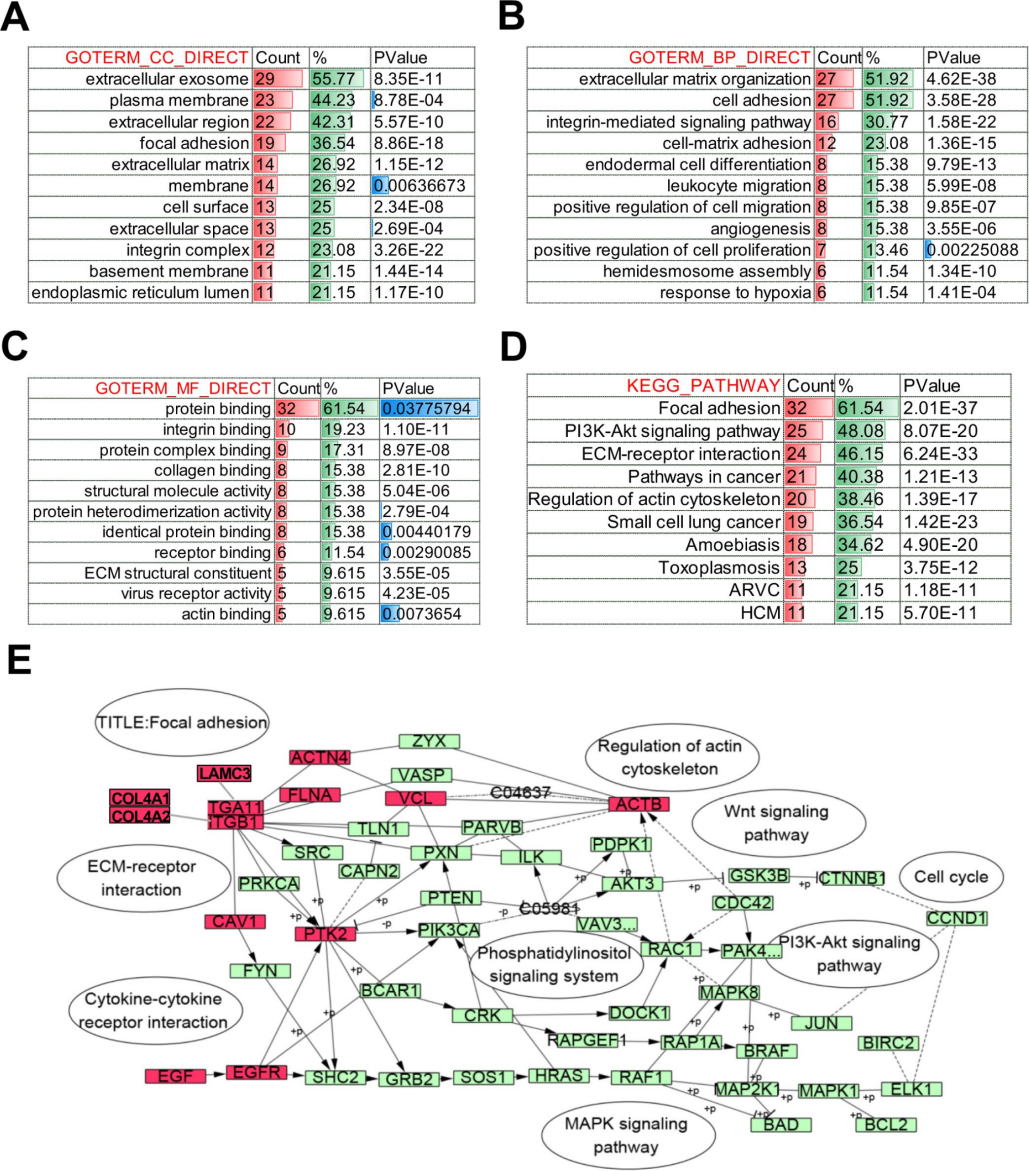
**Biological function of *COL4A1* and *COL4A2* signaling network alterations in HCC.** The histogram showing the biological function of the top 50 most frequently altered neighbor genes of *COL4A1* and *COL4A2* in HCC. (**A**) Cellular components. (**B**) Biological processes. (**C**) Molecular functions. (**D**) KEGG pathway analysis. (**E**) Network showing the KEGG pathway of Focal adhesion. The altered genes are highlight in red.

Further, to identify the altered biological function of *COL4A1* and *COL4A2* networks in HCC, the most frequently altered neighbor genes (a total of 50) were collected and evaluated by analyzing GO and KEGG in the DAVID database. The *COL4A1*/*COL4A2* neighboring genes with alteration frequencies >10% (16 out of a total of 94) in HCC were listed in Figure 4D and Table 1. Similar to *COL4A1* and *COL4A2*, mRNA up-regulation and amplification were the main alteration types for a majority of these neighboring genes. The most frequently altered neighbor genes of *COL4A1*/ *COL4A2* were *PTK2* (46.4%), *ACTN2* (24.1%), and *THBS3* (20.1%). Analysis of significantly enriched GO results indicated that the proteins encoded by these genes localized primarily to the extracellular exosome, plasma membrane, and extracellular region (Figure 5A), where they were mainly involved in the extracellular matrix organization, cell adhesion, and integrin-mediated signaling pathway (Figure 5B). These proteins also served as structural constituents of protein binding, integrin binding, protein complex binding, and collagen binding (Figure 5C). Moreover, KEGG analysis showed enrichment in focal adhesion, PI3K-Akt pathway, ECM-receptor interaction, and pathway in cancer (Figure 5D). Thus, the biological interaction network of *COL4A1* and *COL4A2* alterations is involved in the extracellular matrix (ECM) and several ECM-receptor activated pathways.

### The biological function of *COL4A1* and *COL4A2* in HCC

**Figure 6:**
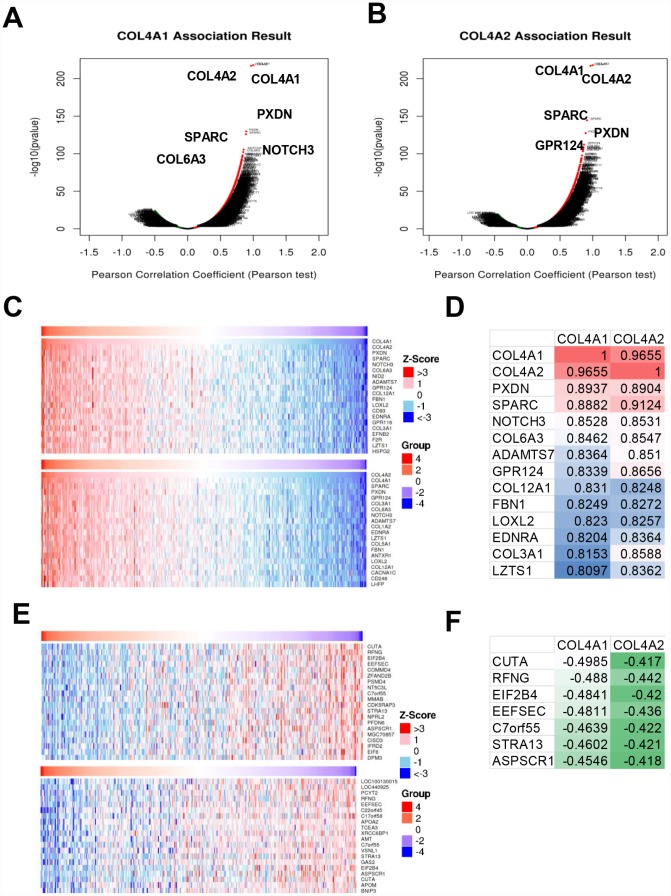
**The genes correlated with *COL4A1* and *COL4A2* and differentially expressed in HCC (LinkedOmics).** (**A**, **B**) The volcano plot showing the genes correlated with *COL4A1* and *COL4A2* and differentially expressed in HCC. (**C**, **E**) The heatmap showing the top 20 genes positively or negatively correlated with *COL4A1* or *COL4A2*. (**D**, **F**) The same correlated genes for *COL4A1* and *COL4A2* in top 20 positively or negatively correlated genes.

**Figure 7:**
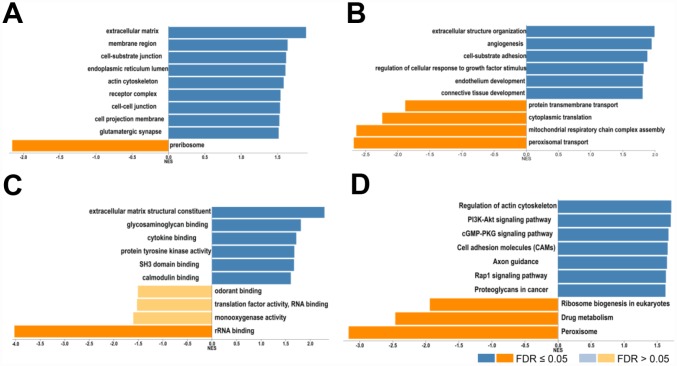
**Biological function of *COL4A1* and *COL4A2* correlated genes in HCC.** GO term and KEGG analysis by GSEA were conducted to clarify the biological function of *COL4A1* and *COL4A2* correlated genes. (**A**) Cellular components. (**B**) Biological processes. (**C**) Molecular functions. (**D**) KEGG pathway analysis. The column represents the Normalized Enrichment Score (NES), and the color of the column represents the FDR.

Next, the genes which correlated with *COL4A1* and *COL4A2* and differentially expressed in HCC were collected and analyzed by LinkedOmics to further examine the biological function of *COL4A1* and *COL4A2* in HCC. *COL4A1* displayed a positive correlation with 7111 genes and negative with 3128 genes; whereas 6680 genes were positively correlated with *COL4A2*, whereas 2714 genes in the opposite (FDR < 0.01, Figure 6A and 6B). The top 20 most positively and negatively correlated genes for *COL4A1* and *COL4A2* were exhibited in the heatmap (Figure 6C–6F). This result implied a similar effect of *COL4A1* and *COL4A2* in the transcriptome. Specially, *COL4A1*/*COL4A2* were highly correlated with PXDN (r = 0.8937 / 0.8904) and SPARC (r = 0.8882 / 0.9124), reflecting significant changes in the extracellular matrix of HCC (Figure 6D). Further, GO term analysis indicated that the *COL4A1* and *COL4A2* correlated genes were located prominently in the extracellular matrix, membrane region, and cell-substrate junction, where they served as structural constituents in the extracellular matrix. They also involved in extracellular structure organization, angiogenesis, and cell-substrate adhesion (Figure 7A–7C). Moreover, KEGG analysis result showed that these genes took part in activating actin cytoskeleton, PI3K-Akt, cGMP-PKG, and cell adhesion pathway (Figure 7D). Thus, these results further demonstrated that the biological interaction network of *COL4A1* and *COL4A2* is involved in ECM-receptor activated pathways.

### *COL4A1* and *COL4A2* network of kinase, miRNA, or transcription factor targets in HCC

**Figure 8:**
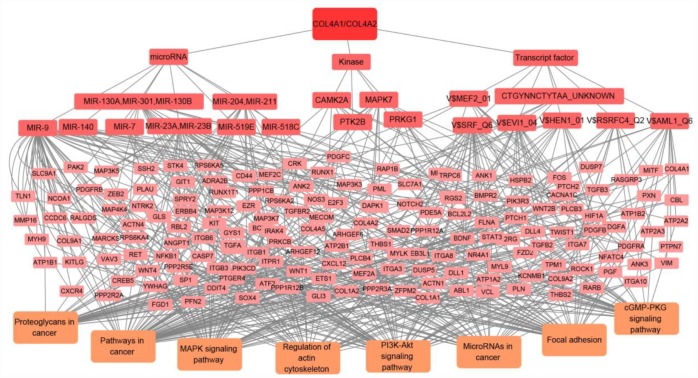
**The transcript factor, microRNA, and kinase targets network of COL4A1 and COL4A2 in HCC (LinkedOmics).** COL4A1 and COL4A2 may involve in hepatocarcinogenesis by regulating the transcript factors-target networks (V$SRF_Q6, V$RSRFC4_Q2, CTGYNNCTYTAA_UNKNOWN, V$MEF2_01, V$AML1_Q6, V$HEN1_01, V$EVI1_04), the kinase-target networks (PRKG1, PTK2B, MAPK7, and CAMK2A), and the microRNA-target networks (MIR-140, MIR-204/MIR-211, MIR-7, MIR-23A/MIR-23B, MIR-130A/MIR-301/MIR-130B, MIR-519E, MIR-518C, and MIR-9).

To discover the specific targets network of *COL4A1* and *COL4A2* in HCC, the most correlated kinases, miRNA, and transcription factors were collected and analyzed by GSEA. As summarized in Table 2, for *COL4A1* and *COL4A2* in HCC, the most correlated kinase-targets network were PRKG1, PTK2B, MAPK7, and CAMK2A; the most correlated microRNA-targets network were MIR-140, MIR-204/MIR-211, MIR-7, MIR-23A/MIR-23B, MIR-130A/MIR-301/MIR-130B, MIR-519E, MIR-518C, and MIR-9; the most correlated transcript factor-target networks were V$SRF_Q6, V$RSRFC4_Q2, CTGYNNCTYTAA_UNKNOWN, V$MEF2_01, V$AML1_Q6, V$HEN1_01 and V$EVI1_04 (v7.4 TRANSFAC). The target genes of these transcript factors, kinases, and microRNAs in HCC were listed in Supplementary Table 1. Furthermore, KEGG analysis showed that these genes involved in pathways in cancer, PI3K-Akt signaling pathway, focal adhesion, MAPK signaling pathway, regulation of actin cytoskeleton, microRNAs in cancer, proteoglycans in cancer, and cGMP-PKG signaling pathway (Figure 8). Thus, *COL4A1* and *COL4A2* may involve in hepatocarcinogenesis by activating the above-mentioned transcript factors-target networks, the kinase-target networks, and the microRNA-target networks.

**Table 2:** The transcript factor, microRNA, and kinase regulatory network of COL4A1 and COL4A2 in HCC (LinkedOmics).

**Type**	**Gene Set**	**Size**	**Leading edge number**	**Enrichment Score (ES)**	**Normalized Enrichment Score (NES)**	**FDR**
Transcript factor	V$SRF_Q6	231	94	0.55044	1.6597	0.00565
	V$RSRFC4_Q2	198	83	0.51218	1.5405	0.00674
	CTGYNNCTYTAA_UNKNOWN	81	30	0.5617	1.6209	0.00698
	V$MEF2_01	134	51	0.54747	1.6178	0.00707
	V$AML1_Q6	248	110	0.49829	1.5057	0.00736
	V$HEN1_01	182	70	0.50724	1.5007	0.00739
	V$EVI1_04	218	101	0.49922	1.5135	0.00746
	GKCGCNNNNNNNTGAYG_UNKNOWN	52	17	-0.20659	-1.2159	0.44429
	V$PPARG_01	40	14	-0.21978	-0.96831	0.76375
microRNA	AAACCAC, MIR-140	100	55	0.51847	1.5346	0.01111
	ACCAAAG, MIR-9	458	210	0.49907	1.5234	0.01114
	AAAGGGA, MIR-204, MIR-211	211	102	0.51606	1.5365	0.01145
	GTCTTCC, MIR-7	149	61	0.51346	1.5208	0.01147
	AATGTGA, MIR-23A, MIR-23B	389	198	0.50816	1.5423	0.01158
	TTGCACT, MIR-130A, MIR-301, MIR-130B	365	220	0.5123	1.5697	0.01165
	GGCACTT, MIR-519E	113	72	0.5321	1.5528	0.01183
	TCCAGAG, MIR-518C	138	65	0.53458	1.5672	0.01187
Kinase	Kinase_PRKG1	30	15	0.7136	1.9136	0
	Kinase_PTK2B	6	3	0.87902	1.7424	0.03337
	Kinase_MAPK7	30	13	0.64928	1.7218	0.03881
	Kinase_CAMK2A	52	23	0.59432	1.6769	0.04961

### COL4A2 overexpression was positively correlated with shorter progression-free survival in HCC patients

**Figure 9:**
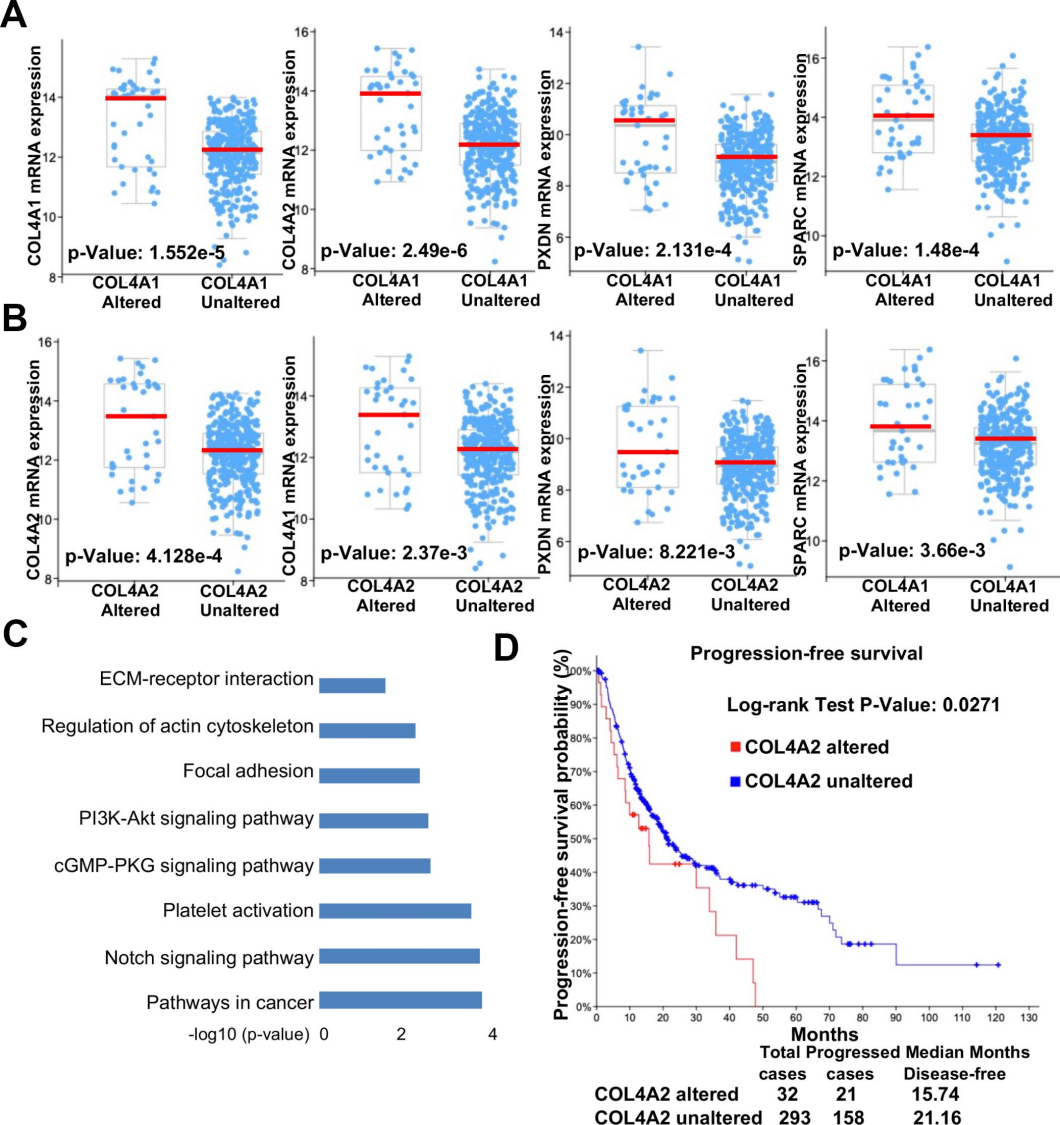
**Collagen VI mutation and overexpression positively correlated with the progression-free survival of HCC patients.** (**A**–**B**) Scatter plot comparison of mRNA levels (COL4A1, COL4A2, PXDN, and SPARC) between genomic altered and unaltered groups (**A**: COL4A1, **B**: COL4A2). (**C**) Column plot illustration of activated signaling pathways in COL4A1/COL4A2 genomic altered HCC samples. (**D**) Kaplan-Meier survival analysis of HCC patients with or without altered COL4A2 DNA sequences.

To demonstrate the direct association of COL4A1 and COL4A2 with HCC, the COL4A1 and COL4A2 genomic altered or unaltered HCC patients were collected to evaluate their clinical features. As shown in Figure 9A, the mRNA level of *COL4A1* and its associated genes, such as *COL4A2*, *PXDN*, and *SPARC*, were higher in the COL4A1-altered group than in the COL4A1-unaltered group. Similarly, the *COL4A2* and its correlated genes, such as *COL4A1*, *PXDN*, and *SPARC*, were overexpressed in the COL4A2-altered patients compared to the COL4A2-unaltered patients (Figure 9B). Additionally, the overexpressed COL4A1 or COL4A2 could activate pathways in cancer including notch, platelet activation, cGMP-PKG, PI3K-Akt, focal adhesion, actin cytoskeleton, and ECM-receptor interaction (Figure 9C), which was consistent with the above biological pathways activated by COL4A1 and COL4A2 (Figure 7D). Furthermore, compared to the COL4A2-unaltered group, the COL4A2-altered group was significantly associated with shorter progression-free survival (P = 0.0271, Figure 9D). Therefore, it strongly suggested that COL4A2 overexpression might promote HCC progression after initial treatment.

## DISCUSSION

Tumor microenvironment including inflammatory cells, stroma, and the extracellular matrix is critically important for tumor initiation and progression [15]. COL IV is a major structural component of the tumor microenvironment, which was steep increased during liver preneoplastic lesion, such as fibrosis and cirrhosis [18]. Thus, COL IV may involve initiating hepatocarcinogenesis. To investigate the different role of each isoform of COL IV in hepatocarcinogenesis, the HCC expression profile was analyzed using public sequencing data in GEO and TCGA, in which among the six COL IV isoforms, only *COL4A1* and *COL4A2* was significantly upregulated from preneoplastic lesions (cirrhosis and dysplasia) to HCC. Then, the *COL4A1* and *COL4A2* network genomic alterations, biological function, and regulatory network were further analyzed to provide deep insights into the function of COL IV in hepatocarcinogenesis.

In this study, the transcriptional levels of *COL4A1* and *COL4A2* in approximately 500 clinical samples from two GEO datasets and one TCGA dataset were significantly increased in cirrhosis and HCC. Both *COL4A1* and *COL4A2* were found in the top 5% of the over-expression-gene-rank of liver cirrhosis and the top 3% of HCC. Moreover, they were strongly correlated with clinic pathological features of patients with HCC based on ethnicity, gender, age, tumor grade, and disease stages. Thus, *COL4A1* and *COL4A2* may be helpful in the diagnose of HCC. As the detection of early HCC is still not systematic, more than 60% of patients are diagnosed with advanced HCC [22]. On the other hand, HCC patients have a better prognosis with a 5-year survival rate of more than 70% if diagnosed at an early stage [23]. Therefore, extensive researches have been conducted on identifying the makers for early HCC, many markers, such as AFP-L3 [24], DCP [25], GPC3 [26, 27], OPN [28], GP73 [29], SCCA [30], annexin A2 [31], suPAR [32], MDK [33], AXL [34], and TRX [35] were screened and undergoing further assessment in phase II studies. Considering the high heterogeneity in HCC patients, it is necessary to combine multiple markers for the detection of early HCC. Thus, combining the above markers with *COL4A1* and *COL4A2* will improve the diagnose reliability of early HCC.

The accumulation of cooperative genomic alterations enables the cells to grow rapidly and develop into tumors [36, 37]. The mRNA up-regulation and amplification were the most genomic alteration types for *COL4A1*/*COL4A2* network genes in HCC. Among the altered genes, PTK2, encoding a cytoplasmic protein tyrosine kinase which was found concentrated in the focal adhesions, was the most frequently altered in HCC (46.4%). Upon activation, PTK2 regulates a variety of cellular functions, including cell adhesion, survival, proliferation, and migration [38–41]. Multiple studies demonstrated that PTK2 was overexpression and hyperphosphorylation in HCC [42–44], and the recent studies described that PTK2 activated CSC properties and tumorigenicity of HCC cells, leading to HCC recurrence and sorafenib resistance [44]. Therefore, *COL4A1* and *COL4A2* may mediate the initiation and progression of HCC by activating PTK2. Moreover, the functional analysis of altered genes of *COL4A1*/*COL4A2* network showed that these genes involved in PI3K/Akt signaling pathway, which was the one classical downstream signal of PTK2. Accumulated evidence showed that overactivated PI3K/Akt/mTOR signaling pathway frequently occurs in HCC, which was highly correlated with poor prognosis and poor overall survival [45, 46]. All these evidences indicated that activation of PTK2-PI3K/Akt/mTOR pathway by *COL4A1* and *COL4A2* may contribute to hepatocarcinogenesis. This result was further confirmed by the significant correlation between COL4A2 overexpression and shorter progression-free survival. However, due to the present insufficient data between *COL4A1* dominant mutations and progression-free survival, the correlation between COL4A1 and hepatocarcinogenesis needs to be explored further.

Furthermore, to get a systematic regulatory network of *COL4A1*/*COL4A2* in HCC, the GSEA was conducted to identify the networks of transcription factors, kinases, and miRNAs. The above networks showed a strong correlation between *COL4A1*/*COL4A2* and PI3K/Akt, cGMP-PKG, MAPK and other pathways in cancer. Among the networks of transcription factors, SRF was the most significant one with the highest enrichment score. Several studies indicated that dysregulated SRF could trigger HCC formation and progression, and SRF was also involved in EMT transition which led to sorafenib resistant in HCC [47–50]. Thus, *COL4A1* and *COL4A2* may involve in hepatocarcinogenesis via SRF transcript factor. Moreover, the other kinase networks including PRKG1, MAPK7, and CAMK2 were associated with *COL4A1* and *COL4A2*, which may also be the potential targets for HCC treatment. However, the studies focused on the relationship between these kinases and HCC are still insufficient, and further studies are needed. Among the networks of miRNAs, MIR-9, MIR-7, MIR-140, and MIR-204 displayed a role in inhibiting the proliferation, progression, metastasis, sorafenib resistant of HCC [51–55], which may be promising targets for HCC management.

This study analyzed the expression and regulatory network of COL IV in hepatocarcinogenesis. Our finding suggested that the increased expression of *COL4A1* and *COL4A2* may involve in HCC initiation and progression by activating PTK2–PI3K/Akt signaling pathway. Last but not least, SRF, a tumor-associated transcription factor, may also involve in hepatocarcinogenesis induced by *COL4A1* and *COL4A2*.

## MATERIALS AND METHODS

### Ethics statement

This study was approved by the Academic Committee of No. 2 Affiliated Hospital, Guangzhou Medical University, Guangzhou, China, and the investigation was conducted according to Declaration of Helsinki principles. All the datasets were collected from the publishing literature, so all written informed consent was obtained.

### Analysis of gene expression profile in preneoplastic lesions and HCC using GEO data

To analyze the expression profile of six COL IV isoforms in preneoplastic lesions and HCC, GEO datasets (access #: GSE14323 and GSE6764) were downloaded, evaluated, and normalized by different R packages, such as GEOquery and limma [56, 57]. The expression profile was mapped by Graphpad Prism [58]. The mRNA levels of six COL IV isoforms in preneoplastic lesions and HCC tissues were compared with that in the normal tissues, using the Student’s t-test to calculate p-value. P ˂ 0.05 were considered different significantly (*, P < 0.05, **, P < 0.01, ***, P < 0.001, ****, P < 0.0001).

### Oncomine analysis

The mRNA expression fold change and Over-expression Gene Rank of *COL4A1* and *COL4A2* in HCC were analyzed by using the Oncomine database. Oncomine (www.oncomine.org) is the current world’s largest microarray database with 715 datasets (86733 samples) [59, 60]. The datasets used here were Mas Liver and Wurmbach Liver, which matched the GSE14323 and GSE6764 datasets in GEO [61, 62]. The mRNA levels of *COL4A1* and *COL4A2* in preneoplastic lesions (cirrhosis) and HCC tissues were compared with that in the normal tissues. Student’s t-test was performed to generate a p-value.

### UALCAN analysis

The relationship between the mRNA levels of *COL4A1*/*COL4A2* and the pathological clinic features of patients with HCC on ethnicity, gender, age, tumor grade, and disease stages was analyzed by using UALCAN. UALCAN (http://ualcan.path.uab.edu) is a web portal to facilitate gene expression analysis of cancer subgroups and cancer survival analyses [63]. The mRNA levels of *COL4A1* and *COL4A2* in HCC samples were compared with that in the normal tissues. P ˂ 0.05 were considered different significantly (*, P < 0.05, **, P < 0.01, ***, P < 0.001, ****, P < 0.0001). Student’s t-test was performed to generate a p-value.

### GEPIA (Gene Expression Profiling Interactive Analysis) database

The expression profile of six COL IV isoforms in HCC was further examined by using GEPIA database. GEPIA web provides a server to analyze the gene expression profiling between cancer and normal tissues [64]. The significance test method was one-way ANOVA, using disease state (Tumor or Normal) as variable for calculating differential expression.

### cBioPortal for cancer genomics

The cooperative genomic alterations of *COL4A1* and *COL4A2* network were analyzed by using cBioPortal. The cBioPortal for Cancer Genomics (http://cbioportal.org) is an integrated website for analyzing complex cancer genomics and clinical profiles [37]. The liver hepatocellular carcinoma (TCGA, Provisional) including 373 samples with mRNA data was selected for further analysis. The genomic profiles included mutations, putative CNA, and mRNA expression. The overview of *COL4A1* and *COL4A2* genomic alterations was shown in the tab OncoPrint. The 50 most frequently altered neighbor genes of *COL4A1* and *COL4A2* were visualized in the tab Network. GO and KEGG pathway of the 50 most frequently altered neighbor genes were analyzed by using DAVID [65].

### LinkedOmics analysis

The correlated genes of *COL4A1* and *COL4A2* in HCC were analyzed by using LinkedOmics. The LinkedOmics database (http://www.linkedomics.org/login.php) is a web-portal for multi-omics and clinical data analysis of 32 cancer types with 11158 samples from TCGA [66]. The genes correlated with *COL4A1* and *COL4A2* in HCC were visualized in the LinkFinder module. The GO (CC, BP and MF), KEGG pathways, kinase-target, miRNA-target and transcription factor-target analysis of the *COL4A1* and *COL4A2* correlated genes were visualized in the LinkInterpreter module.

## Supplementary Material

Supplementary Figure 1

Supplementary Table 1
