# Supplementary material for: The correlation and role analysis of COL4A1 and COL4A2 in hepatocarcinogenesis
Source: Aging (Albany NY). 2020 Jan 5;12(1):204–23. doi: 10.18632/aging.102610 (PMC6977693; doi:10.18632/aging.102610)
Supplement: Supplementary Figure 1 [file aging-12-102610-s002..pdf]

SUPPLEMENTARY FIGURE

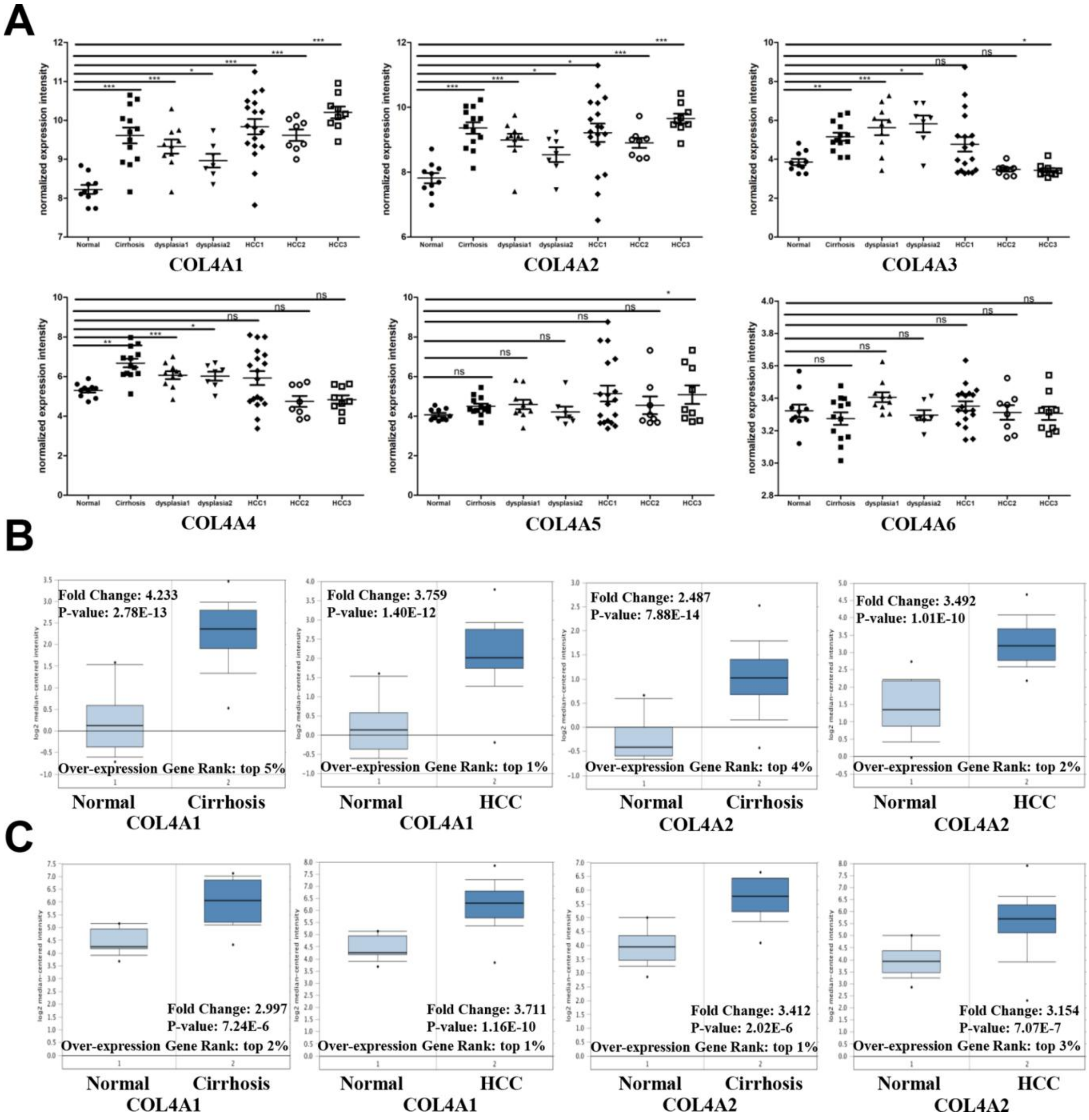

Supplementary Figure 1. COL4As expression in hepatocellular carcinoma (HCC). The transcription levels of *COL4A1* and *COL4A2* were significantly upregulated in preneoplastic lesions (cirrhosis and dysplasia) and HCC tissues compared to normal tissues. (**A**) Dot plot showing the COL4As mRNA levels in GEO dataset (GSE6764). Normal (n=10): normal liver; Cirrhosis (n=13): cirrhotic liver tissue; dysplasia1 (n=10): low-grade dysplastic liver tissue; dysplasia2 (n=7): high-grade dysplastic liver tissue; HCC1(n=18): early HCC; HCC2 (n=8): advanced HCC; HCC3 (n=9): very advanced HCC. Data are mean ± SD. *, P < 0.05, **, P < 0.01, ***, P < 0.001 (Student's t-test). (**B**) Box plot showing the fold change of *COL4A1* and *COL4A2* in preneoplastic lesion (cirrhosis) and HCC tissues compared to normal tissues in GSE14323 dataset (Mas Liver, Oncomine). (**C**) Box plot showing the fold change of *COL4A1* and *COL4A2* in preneoplastic lesion (cirrhosis) and HCC tissues compared to normal tissues in GSE6764 dataset (Wurmbach Liver, Oncomine). Student's t-test was performed to generate a p-value.
