# Supplementary material for: The correlation and role analysis of COL4A1 and COL4A2 in hepatocarcinogenesis
Source: Aging (Albany NY). 2020 Jan 5;12(1):204–23. doi: 10.18632/aging.102610 (PMC6977693; doi:10.18632/aging.102610)
Supplement: Supplementary Table 1 [file aging-12-102610-s001..pdf]

SUPPLEMENTARY TABLE

Supplementary Table 1. Significantly enriched transcript factor, microRNA, and kinase networks of COL4A1 and COL4A2 in hepatocellular carcinoma (LinkedOmics).

Geneset	LeadingEdgeGene
V$SRF_Q6	COL1A2;COL1A1;FLNA;VCL;MYL9;KCNMB1;PLN;PPP1R12A;RARB;NFATC4;ZEB1;BDNF;SLC7A1;THBS1;FOS;ABL1;ACTN1;PTCH1;PPP2R3A;NR4A1;DUSP5;ITGA7;ATP1A2;MYLK;PLCB3
V$RSRFC4_Q2	THBS2;ZFPM2;TGFB3;RASGRP3;TWIST1;PDGFRA;ATP2A3;ATP1B2;MITF;PIK3R3;PTCH1;NR4A1;ITGA7
CTGYNNCTYTAA_UNKNOWN	MRVI1;MECOM;ITGA3;IRAK4
V$MEF2_01	ZFPM2;MEF2C;DLL4;PDGFRA;ANK3;FOS;ITGB6;ITGA7
V$AML1_Q6	COL4A1;COL4A2;VIM;PGF;CBL;ITGA10;ANK3;NOTCH2;ATP2A2;NR4A1;TPM1;PXN;COL9A2;PTPN7
V$HEN1_01	PDGFB;DLL4;CREB3L1;DLL1;HIF1A;BDNF;ANK1;PDGFA;ROCK1;ANK2;DUSP7;BCL2L2;MAP3K3;STAT3
V$EVI1_04	CACNA1C;HSPB2;TWIST1;ITGA8;TGFB2;WNT2B;BMPR2;FZD2;NFATC4;BDNF;NOTCH2;PTCH2;PTCH1;TPM1;IL2RG;PDGFC
PRKG1	MRVI1;ADCY3;PRKG1;PDE5A;TRPC6;ARHGEF6;RGS2;RAP1B
PTK2B	NOS3
MAPK7	MEF2C;ETS1;RPS6KA2;HIF1A;RUNX1;NFKB1;FOS;SGK1;MEF2A;DAPK1;PML;NR4A1
CAMK2A	VIM;ADCY3;ETS1;FLNA;PLN;ITGB1;CD44;PLCB3;SMAD2
MIR-140	CACNA1C;PDGFRA;ANK2;BCL2L2;SOX4;ACTN4;GYS1;E2F3;GIT1;WNT1
MIR-9	COL4A2;PDGFRB;SLC9A1;MMP16;CXCR4;TLN1;MYH9;NFKB1;RALGDS;CREB5;NOTCH2;RET;ANK2;PIK3R3;COL9A1;VAV3;WNT4;KITLG;MAP3K3;CCDC6;NCOA1;PAK2;PDGFC;RPS6KA4;ATP1B1
MIR-204,MIR-211	NTRK2;ITPR1;BCL2;ANGPT1;TGFBR2;CREB5;WNT4;KITLG;EZR;SOX4;MAP3K3;ATF2;RPS6KA5;ATP2B1;YWHAG
MIR-23A,MIR-23B	MEF2C;RUNX1T1;PLAU;PLCB4;PPP1R12A;CXCL12;PTGER4;ZEB1;PPP2R5E;GLS;SLC7A1;TGFBR2;TGFA;SGK1;KITLG;MAP3K5;SSH2;SPRY2;MAP3K3;PPP1CB;CCDC6;CRK;DUSP5;ADRA2B;NCOA1;MAP4K4;COL4A5;MARCKS;YWHAG;STK4;CASP7
MIR-519E	CXCR4;DLL1;PPP2R2A;ARHGEF12;MYLK;SP1;FGD1;MAP3K7;RBL2
MIR-7	GLI3;DDIT4;PRKCB;PIK3CD;SP1;ERBB4;PFN2
MIR-518C	DLL4;PPP1R12B;TWIST1;ANK2;ITGB3;ATP2A2;SPRY2
MIR-130A,MIR-301,MIR-130B	ZFPM2;ZEB2;MAP3K12;KIT;DLL1;ITPR1;PDGFRA;WNT2B;BMPR2;TGFBR2;CREB5;ARHGEF12;SOX4;NCOA1;ERBB4;WNT1
